# RETRACTION: Study on the Biological Characteristics of CD133^+^ Cells Interfered by RNA Interference in Gastric Cancer

**DOI:** 10.1155/isr3/9806262

**Published:** 2025-02-27

**Authors:** International Scholarly Research Notices

**RETRACTION**: J. Yu, S. Wang, J. Wu, R. Lu, X. Ni, C. Cai, and B. Jiang, “Study on the Biological Characteristics of CD133^+^ Cells Interfered by RNA Interference in Gastric Cancer,” *International Scholarly Research Notices* 2014, no. 1 (2014): 329519, https://doi.org/10.1155/2014/329519.

The above article, published online on 19 March 2014 in Wiley Online Library (http://wileyonlinelibrary.com), has been retracted by John Wiley and Sons Ltd. The retraction has been agreed upon following an investigation into concerns raised by a third party, which revealed inappropriate image panel duplications within the article depicting different experimental conditions (Figures 3c, 7c, 8a, and 9a). Thus, the publisher has lost confidence in the presented data and consider the conclusions of this manuscript substantially compromised. The authors and their institute were informed about the concerns, but they remained unresponsive.

